# 500 Return To Work After A Burn Injury: Even The Small Burns Need Attention

**DOI:** 10.1093/jbcr/irac012.131

**Published:** 2022-03-23

**Authors:** Melissa Carmean, Victor C Joe, Nicole O Bernal, Kimberly Burton, Theresa L Chin

**Affiliations:** UCI Health, Orange, California; UCI Health Regional Burn Center, Orange, California; The Ohio State University Wexner Medical Center, Columbus, Ohio; University of California Irvine, Orange, California; University of California Irvine, Orange, California

## Abstract

**Introduction:**

An important part of burn recovery and reintegration involves the return to meaningful employment. Studies have determined that the workman’s compensation burn patient faces multiple barriers to return to work (RTW). Factors that increase time to RTW include increased TBSA burns, full thickness depth and grafting, burns to the hands and feet, and increased age. We hypothesize that patients with grafted burns, hand and feet burns, or psychological disorders following injury take longer to return to work.

**Methods:**

A retrospective analysis was performed of burn patients seen in the outpatient burn clinic with work related injuries equal to or less than 5% TBSA, from 11/1/2016 to 5/31/2021. In addition to demographic data, also collected were locations of burns, dates of RTW, and positive PTSD and depression symptoms on screening.

Medians with interquartile ranges are reported due to nonnormal distribution, Wilcoxon Mann Whitney tests were performed using StataCorp. burn patients seen in the outpatient burn clinic with work related injuries.

**Results:**

Of 118 patients, more injuries occurred in males (89%) than females with a median age of 36.5 years (IQR 26-49). The median TBSA was 1% (IQR .025-1.5) and the median time to return to work was 34 days (IQR 16-75). The 21 patients that were grafted took significantly longer to return to work (median 107.5 days; IQR 60-199.5) compared to 97 patients that were not grafted (median 28 days; IQR 13-59; p=0.0000]. Presence of a hand/foot burn trended towards increased time to RTW but was not significant, median 37 days (IQR 19-81) vs 26 days (IQR 14-61; p =.0306).

Patients with PTSD and depression (n=16) also took longer to return to work (Median 66; IQR 33-89.5) compared to patients (n=90) that showed no symptoms on screening (median 30.5; IQR 14-72; p=0.032).

**Conclusions:**

The increased RTW for grafted, hand and foot burns is supported in the current research, however the addition of PTSD and depression and its interplay with RTW has not yet been fully vetted in determining return to work readiness. Within our population, positive screens for these psychological stressors profoundly affected their ability to return to employment. This study continues to validate the need for early screening and intervention in our population and the need to intervene as early as possible with psychological support services.

